# Integrating care for patients and populations: developing a national strategy for integrated health and social care in England

**Published:** 2012-09-04

**Authors:** Nick Goodwin

**Affiliations:** The King’s Fund, UK

**Keywords:** integrated care, UK, health reforms, measurable goals

## Abstract

This workshop examines the rise of integrated care as a central component of the UK Government’s current reforms to its health and social care system. In particular, the workshop presents the key debates and conclusions from work undertaken by The King’s Fund and the Nuffield Trust [1]—two key health policy ‘think tanks’ in the UK—in direct support of the UK Government’s emerging legislation and strategy. This work included a review of the evidence-base for integrated care; workshops and interviews with managers and clinicians across health and social care and at the forefront of integrated care delivery; and meetings with policy-makers to discuss and refine key messages. The workshop will examine the report’s conclusions and its ten key recommendations for the effective development of integrated care in England. In particular, it will examine how a clear, ambitious and measurable set of goals are required if the experiences and outcomes to patients and service users is to be improved.

Powerpoint presentation available at http://www.integratedcare.org at congresses – San Marino – programme.
